# CD8^+^ T Cells Specific to Apoptosis-Associated Antigens Predict the Response to Tumor Necrosis Factor Inhibitor Therapy in Rheumatoid Arthritis

**DOI:** 10.1371/journal.pone.0128607

**Published:** 2015-06-10

**Authors:** Alessandra Citro, Rossana Scrivo, Helene Martini, Carmela Martire, Paolo De Marzio, Anna Rita Vestri, John Sidney, Alessandro Sette, Vincenzo Barnaba, Guido Valesini

**Affiliations:** 1 Dipartimento di Medicina Interna e Specialità Mediche, Sapienza Università di Roma, Viale del Policlinico 155, 00161 Rome, Italy; 2 Dipartimento di Sanità Pubblica e Malattie Infettive, Sapienza Università di Roma, Rome, Italy; 3 La Jolla Institute for Allergy and Immunology, San Diego, California 92121, United States of America; 4 Istituto Pasteur-Fondazione Cenci Bolognetti, 00185 Rome, Italy; University of Nebraska-Lincoln, UNITED STATES

## Abstract

CD8^+^ T cells specific to caspase-cleaved antigens derived from apoptotic T cells (apoptotic epitopes) represent a principal player in chronic immune activation, which is known to amplify immunopathology in various inflammatory diseases. The purpose of the present study was to investigate the relationship involving these autoreactive T cells, the rheumatoid arthritis immunopathology, and the response to tumor necrosis factor-**α** inhibitor therapy. The frequency of autoreactive CD8^+^ T cells specific to various apoptotic epitopes, as detected by both enzyme-linked immunospot assay and dextramers of major histocompatibility complex class I molecules complexed with relevant apoptotic epitopes, was longitudinally analyzed in the peripheral blood of rheumatoid arthritis patients who were submitted to etanercept treatment (or other tumor necrosis factor inhibitors as a control). The percentage of apoptotic epitope-specific CD8^+^ T cells was significantly higher in rheumatoid arthritis patients than in healthy donors, and correlated with the disease activity. More important, it was significantly more elevated in responders to tumor necrosis factor-α inhibitor therapy than in non-responders before the start of therapy; it significantly dropped only in the former following therapy. These data indicate that apoptotic epitope-specific CD8^+^ T cells may be involved in rheumatoid arthritis immunopathology through the production of inflammatory cytokines and that they may potentially represent a predictive biomarker of response to tumor necrosis factor-**α** inhibitor therapy to validate in a larger cohort of patients.

## Introduction

Rheumatoid arthritis (RA) is an autoimmune disease occurring in 0.5% to 1.0% of the adult population worldwide, principally characterized by inflammatory polyarthritis with localized inflammation of joint synovial tissue and progressive destruction of bone and cartilage [1]. Complex interactions among genetic, immunologic, and environmental factors play a role in RA development [1–8].

Both pro-inflammatory innate (e.g., dendritic cells [DCs], macrophages, and neutrophils) and adaptive (e.g., T helper [Th]1, Th17, CD8 T, and B) cells, which can organize into discrete lymphoid aggregates with germinal centers in RA, are strongly involved in initiating and maintaining the disease through the production of autoantibodies and many cytokines that act both in series and in parallel, meaning cascades of action and redundancy [1,2,9–14]. Tumor necrosis factor (TNF)-**α** interleukin (IL)-1 family of cytokines (IL-1**α**, IL-1**β**, IL-18, and IL-33), and IL-6, particularly those produced by activated macrophages (predominantly M1 macrophages, the major effectors of synovitis), exert pro-inflammatory effects mediated by the induction of other pro-inflammatory cytokines, metalloproteinases, free radicals, serine proteases, and aggrecanases [1,2].

As a consequence of the activated inflammatory pathways in the inflamed synovium of RA patients, an enormous number of apoptotic cells results from the rapid turnover of effector T cells undergoing apoptosis after performing their functions. This may further amplify immunopathology [15]. In previous studies, we demonstrated that the proteome of apoptotic T cells includes prominent caspase-cleaved cellular proteins (i.e., fragments cleaved from long-lived proteins that are anchored to cellular structures), such as actin cytoplasmic 1 [(ACTB]), heterogeneous nuclear ribonucleoprotein [(ROK]), lamin B1 [(LAM1]), non-muscle myosin heavy chain 9 [(MYH9]), vimentin [(VIME]), proteasome component C2 [(PSA1)], rho GDP dissociation inhibitor 2 (GDIS), and 60S acidic ribosomal protein P2 (RLA] [16]. In particular, upon phagocytosis of apoptotic T cells by dendritic cells (DCs), caspases within apoptotic cells can cleave fragments from these long-lived proteins, which are then efficiently processed by DCs that ultimately cross-present a high proportion of distinct epitopes in these fragments (apoptotic epitopes [AEs]) via the classical major hisotocompatibility complex (MHC) class I pathway to a wide repertoire of autoreactive CD8^+^ T cells [16–19]. Importantly, apoptotic cells derived from activated T cells retain the expression of CD40 ligand (CD40L) and, in contrast to CD40L^-^ apoptotic cells (e.g., those derived from epithelial cells), can condition CD40^+^ DCs to acquire high capacities to prime or cross-prime autoreactive T cells specific to apoptotic T cell-derived epitopes [20,21]. This finding is supported by the evidence that the proportion of AE-specific CD8^+^ T cells correlated with the proportion of circulating apoptotic CD4^+^ T cells in vivo and with the disease progression in chronic human immunodeficiency virus (HIV) or acute hepatitis C virus (HCV) infections [16,22]. Research has suggested that the emergence and the maintenance of these responses contribute to establishing the phenomenon of chronic immune activation (CIA) and, ultimately, in amplifying the immunopathology in autoimmune diseases, such as multiple sclerosis (MS), through their capacity to produce high levels of inflammatory cytokines [23].

The proof of principle of the pathogenic role of the panoply of cytokines in RA derives from the observation that the administration of available biological therapeutics (subsumed as biological originator [bo] disease-modifying antirheumatic drugs [DMARDs]), blocking tumor necrosis factor (TNF) (adalimumab, certolizumab pegol, etanercept, golimumab, or infliximab), the interleukin (IL)-6 receptor (IL-6R) (tocilizumab), or IL-1 (anakinra), is associated with clinical improvement [24]. Similarly, research has shown that the administration of monoclonal antibodies (mAbs) against activated B cells (rituximab) or the T cell costimulation inhibitor (abatacept) results in parallel clinical improvement [24].

The aims of the present study are to ascertain if CD8^+^ T cells specific for AEs are prominent in RA patients, to distinguish which of them is associated with disease severity, and to determine if they have the potentiality to predict which patients might (or might not) benefit from therapy with TNF-**α** inhibitors.

## Materials and Methods

### Study Population

Sixteen selected HLA-A2^+^ biologic-naïve RA (according to the 1987 American College of Rheumatology [ACR] criteria) patients (female/male [F/M] = 15/1; median age 53 years range 36–69 years; mean disease duration 96.6 months range 6–240 months) who had shown an unsatisfactory response to conventional DMARDs including methotrexate (associated or not associated with other anti-inflammatory/immunosuppressive drugs) and who submitted to subsequent treatment with etanercept were included in the study (S1 Table). Each patient was given a standard dose of 50 mg etanercept per week subcutaneously and followed for clinical parameters. Clinical response was set in the present study as an improvement of the disease activity score using 28 joint counts (DAS28) > 0.6 after 6 months of therapy in accordance with the European League Against Rheumatism (EULAR) response criteria [25]. Of the 16 patients, 9 were responders (Rs) and 7 were non-responders (NRs) (S1 Table and S1 Fig). As a control, six RA patients (female/male [F/M] = 6/0; median age 60.5 years range 48–70 years; mean disease duration 165 months range 24–288 months) submitted to adalimumab were also enrolled: 2 resulted Rs and 4 NRs (S2 Fig). No difference was detected between Rs and NRs in terms of duration of disease, disease activity (as calculated by DAS28-erythrocyte sedimentation rate [ESR] or DAS28-C-reactive protein [CRP], and serum levels of ACPA or rheumatoid factor (RF) before the start of therapy (S1 Table). Twenty-four HLA-A2^+^ healthy donors (HDs) matched for sex and age with patients were also included. The study was conducted in accordance with the principles expressed in the Declaration of Helsinki. The study protocol was approved by the relevant research ethics committee of Sapienza Università di Roma- Azienda Policlinico Umberto I di Roma (n. 3202/15.05.2014). All patients and controls gave written informed consent.

### Synthetic Peptides

Ninety-one synthetic peptides (nonamers or decamers) were prepared according to the sequence of caspase-cleaved proteins (ACTB, ROK, LAM1, MYH9, GDIS, VIME, PSA1, RLA) that had been identified by the proteomic analyses of apoptotic T cells, as previously described (S2–S4 Tables) [16]. All the peptides were synthesized and selected for their capacity to bind the HLA-A2 molecule [16,26].

### Cell Preparations

Peripheral blood mononuclear cells (PBMCs) were isolated by Ficoll density gradient method. Spontaneous apoptosis of T cells from patients was determined by staining fresh PBMCs with fluorescein isothiocyanate (FITC)-labeled Annexin V (BioLegend), propidium iodide (PI) (BioLegend), and allophycocyanin (APC)-labeled anti-CD3 mAb (BioLegend).

### Enzyme-Linked Immunospot Assay

Following stimulation with 12 independent pools of AEs (S2–S4 Tables), PBMCs were tested by enzyme-linked immunospot (ELISPOT) assay [16,22]. Briefly, 96-well millimeter high-affinity plates (Millipore, Bedford, MA) were coated with 10 μg/ml of capture mAb against interferon (IFN)-**γ** (BD Bioscience) at 4°C overnight. Plates were blocked for 2 h with blocking solution (phosphate-buffered saline [PBS] containing 2% bovine serum albumin [BSA]). A total of 1 × 10^5^ PBMCs were added to each well and stimulated for 18 h with peptides. Biotinylated anti-IFN-**γ** diluted to 5 μg/mlin blocking solution was added and incubated for 2 h in 5% CO_2_ at 37°C. Plates were washed, incubated with alkaline phosphatase (AKP)-streptavidin (BD Bioscience), and developed with SIGMAFAST BCIP/NBT (Sigma). The reaction was stopped by rinsing the plates with distilled water. Each well was then examined for positive dots. The number of dots in each well was counted with an ELISPOT reader system (AELVIS reader system). IFN-**γ**-secreting cells were expressed as IFN-**γ** spots per 1 × 10^6^ cells. The IFN-**γ** spot values were subtracted from the background, which was below 10 IFN-**γ** spots in 1 x 10^6^ cells for each test.

### Monoclonal Antibody and Dextramer Staining

PBMCs were incubated with APC-labeled—HLA-A*0201 dextramers complexed to MYH9_478-486_ (QLFNHTMFI), MYH_9741-749_ (VLMIKALEL), VIME_78-87_ (LLQDSVDFSL), VIME_225-233_ (SLQEEIAFL), or ACTB_266-274_ (FLGMESCGI) peptides (Immudex, Copenhagen, Denmark). The incubation was performed in fluorescence-activated cell sorting (FACS) buffer (PBS containing 2% human AB serum) at room temperature for 10 min, followed by washing and further surface staining with FITC-labeled mAb to CD8 (eBioscience), phycoerythrin-cyanine (PeCy)7-labeled mAb to program death (PD)-1 (eBioscience), AlexaFluor700-labeled mAb to CD69, PE-CF594-labeled mAb to HLA-DR, and a cocktail of labeled mAbs and labeled reagents (APC-Cy7-labeled mAbs to CD4, CD14, CD16, CD19, and CD56 [BioLegend]) and Fixable Viability Dye eFluor 780 (eBioscience) (dump channel) for 20 min at 4°C. Dextramer^+^ cells were analyzed within a CD8^+^ cell gate, whereas CD69^+^, HLA-DR^+^, or PD-1^+^ cells were analyzed within dextramer^+^CD8^+^ cells after the exclusion of B cells, monocytes, natural killer T (NKT) cells, NK cells, and CD4^+^ T cells (dump channel). Cells were acquired with LSRFortessa cytometer (Becton Dickinson) and analyzed with FlowJo software version 7.5.5 (Tree Star, San Carlos, CA).

### Intracellular Cytokine Staining

Cytokine production was analyzed by intracellular staining (ICS) assay. PBMCs were incubated with or without the relevant peptides (20 μg/ml) plus anti-CD28 mAb (4 μg/ml) (BD Biosciences) and the Protein Transport Inhibitor Cocktail (Brefeldin A and Monensin; eBioscience), or with the Cell Stimulation Cocktail (phorbol 12-myristate 13-acetate [PMA], ionomycin, brefeldin A, and monensin; eBioscience) as a positive control, for 18 h at 37°C [16,22]. Cells were washed and then stained with APC-labeled—HLA-A*0201 dextramers complexed to corresponding peptides, PeCy7-labeled mAb to CD8 (BioLegend), and the dump channel reagents. Cells were fixed and permeabilized using the BD Cytofix/Cytoperm Fixation/Permeabilization Solution Kit (BD Biosciences) at 4°C for 20 min, rewashed with the BD Perm/Wash buffer (BD Biosciences), and stained with different combinations of AlexaFluor700-labeled IL-17A (BioLegend) and FITC-labeled anti-IFN-**γ** (BioLegend) for 20 min at 4°C. Cells were washed, acquired with the LSRFortessa cytometer, and analyzed with FlowJo software. IL-17, IFN-**γ** or IL-17/IFN-**γ** producing cells were analyzed in CD8^+^dextramer^+^ cells after exclusion of B cells, monocytes, NKT cells, NK cells, and CD4^+^ T cells (dump channel).

### Statistical Analyses

The collected data underwent statistical analysis with GraphPad Prism version 4 software (GraphPad Software). Comparisons between HDs and patients, comparisons in patients at different times, correlations between the different tests performed, and correlations between tests and clinical data were analyzed with the Mann-Whitney test, the Wilcoxon matched pairs test, linear regression, and the Spearman’s correlation, respectively. The significance threshold was set at *P* = 0.05. Receiver operating characteristic (ROC) analysis was performed to assess the predictive power of AE-specific CD8^+^ T cells. The area under the ROC curve (AUC) was calculated along with 95% confidence intervals (CI). In our study, the AUC value indicates the ability of dextramer^+^ T cells to distinguish Rs and NRs.

## Results

### Multispecific CD8^+^ T Cell Responses to AEs

**Fig 1:**
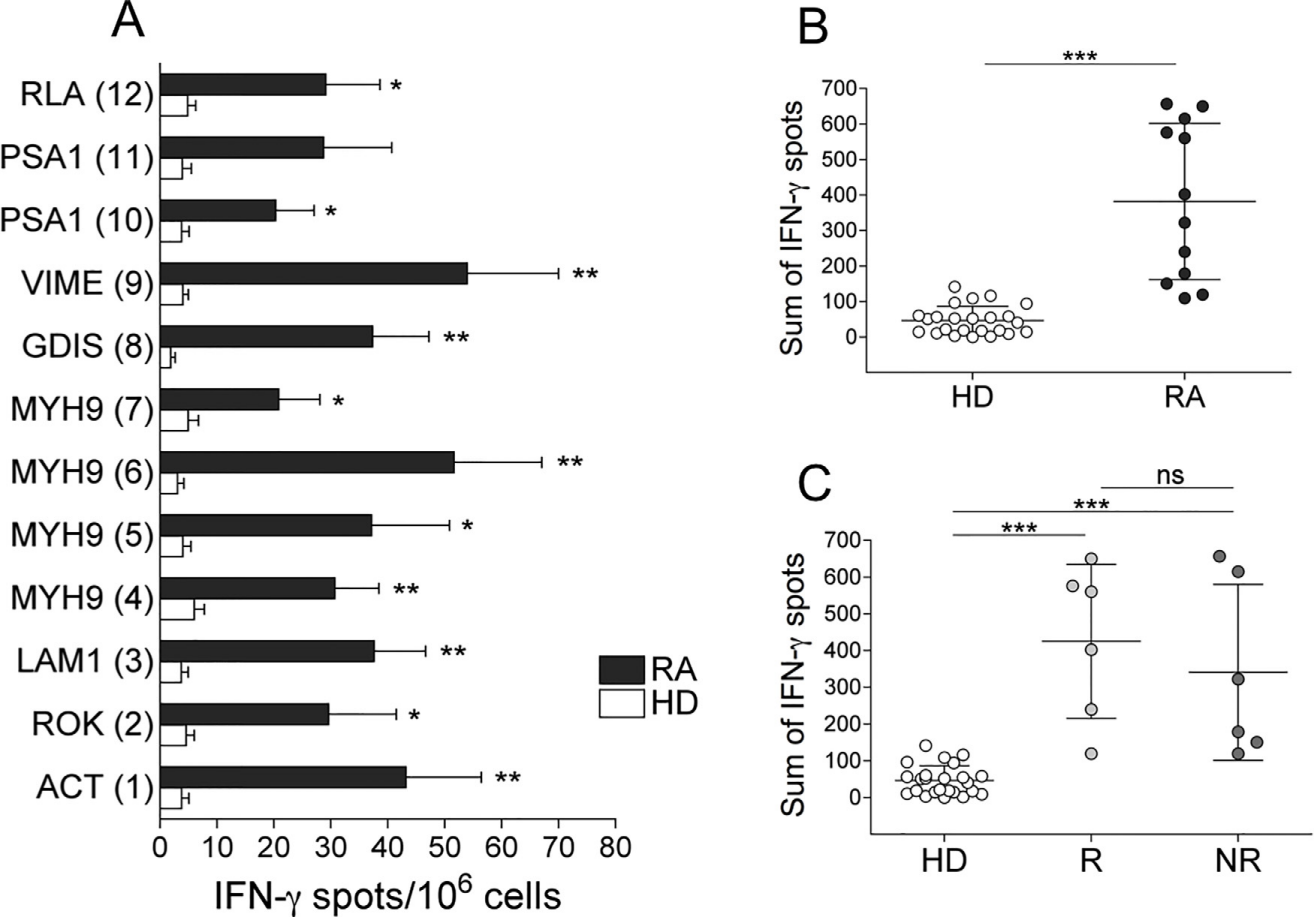
CD8^+^ T cell multispecificity to AEs in HDs and RA patients (A-C). (A) Mean number of IFN-**γ** spots formed by fresh CD8^+^ T_EM_ cells (by ELISPOT assay) in response to 12 pools of AEs (see S2–S4 Tables) in 12 HLA-A2^+^ patients with RA or 24 HLA-A2^+^ HDs. (B) Sum of IFN-**γ** spots formed by fresh CD8^+^ TEM cells in response to all pools (see S2–S4 Tables) of AEs in the single patient or HD. (C) Sum of IFN-**γ** spots formed by fresh CD8^+^ TEM cells in response to all pools (see S2–S4 Tables) of AEs in the single R, NR, or HD. Analyses were performed at time 0 (before the start of therapy). Statistical analysis was performed with the Mann-Whitney test. *P < 0.01; **P < 0.001; ***P < 0.0001. ns = not significant. RLA2 = 60S acidic ribosomal protein P2; PSA1 = proteasome component C2; VIME = vimentin; GDIS = rho GDP dissociation inhibitor 2; Myh9 = non-muscle myosin; LAM1 = Iamin B1; ROK = heterogeneous nuclear ribonucleoprotein K; ACT B = actin cytoplasmic 1.

Initially, we analyzed longitudinally the effector responses based on the capacity of freshly isolated CD8^+^ T cells from either 12 (of the 16) HLA-A2^+^ patients submitted to etanercept or 24 HDs to form IFN-**γ** spots (in an ELISPOT assay) within 4 h to 6 h of contact with 12 pools containing a total of 90 synthetic HLA-A2-binding apoptotic peptides (S2–S4 Tables) [16,22]. These peptides (nonamers or decamers) were prepared according to the sequence of caspase-cleaved proteins (ACTB, ROK, LAM1, MYH9, GDIS, VIME, PSA1, RLA) that had been identified by the proteomic analyses of apoptotic T cells, as previously described [16]. All the peptides were synthesized and selected for their capacity to bind the HLA-A2 molecule (S2–S4 Tables) [16,26]. Therefore, we defined these CD8^+^ cells as effector memory T (TEM) cells on the basis of their capacity to perform their effector functions promptly within a few hours of antigenic stimulus. Each peptide pool was tested in triplicate. The synthetic peptides used were prepared according to the sequence of caspase-cleaved proteins that had been previously identified by the proteomic analyses of apoptotic T cells (e.g., fragments of ACTB, ROK, LAM1, MYH9, GDIS, VIME, PSA1, and RLA) [16,22]. We found that the responses to AEs by IFN-**γ** ^+^CD8^+^ TEM cells were significantly higher and wider in patients than in HDs (Fig 1).

In particular, the median number of IFN-**γ** spots formed by CD8^+^ TEM cells from all RA patients or HDs in response to the single peptide pool (responsiveness) (Fig 1A) and the sum of IFN-**γ** spots formed in response to the total peptide repertoire by a single patient or HD (magnitude) (Fig 1B) were significantly higher in RA patients than in HDs. The HLA-restriction of these responses was demonstrated both by blocking responses with an appropriate anti-class I mAb and by determining that no response was observed in HLA-A2^-^patients (data not shown). No correlation was found between the ELISPOT responses to AEs and the disease activity, as calculated by both DAS28-ESR and DAS28-CRP (data not shown). In addition, no difference in the AE repertoire recognized by IFN-**γ** ^+^CD8^+^ TEM cells was observed between Rs and NRs at the time point tested before the start of therapy (time 0) (Fig 1C).

### AE-Specific CD8^+^ T Cells as Detected by Dextramers Predict the Effect of TNF-α Inhibitor Therapy

**Fig 2:**
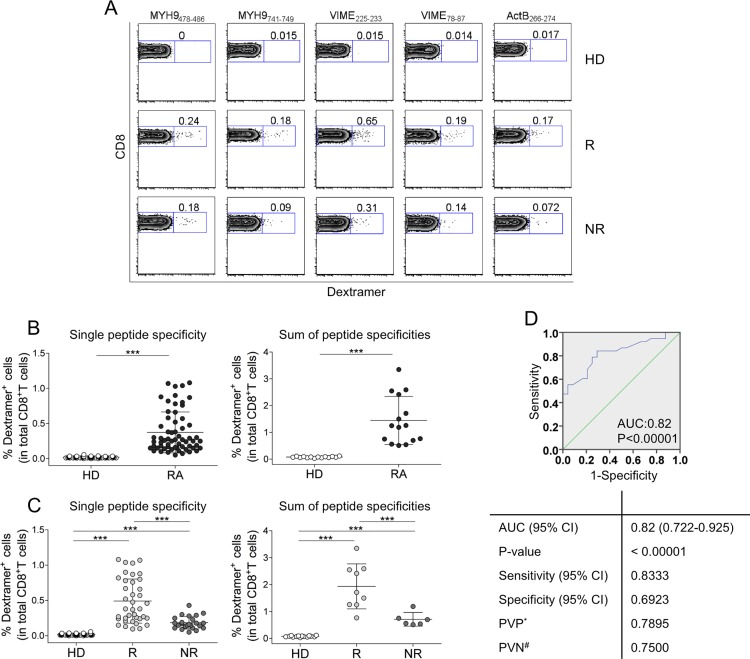
AE-specific CD8^+^ T cells predict RA patients who will (or will not) benefit from etanercept therapy (A-D). (A) Representative flow cytometry analysis of dextramer^+^CD8^+^ T cells specific to AE in an RA patient and an HD. Zebra plot analyses show the percentage of dextramer^+^CD8^+^ cells. (B) Percentage of dextramer^+^CD8^+^ cells in 14 HDs and 15 patients (each symbol represents the percentage of a single dextramer^+^CD8^+^ cell population) (left graph); sum of the percentages of all dextramer^+^CD8^+^ T cells detected in the single HD or patient (each symbol represents the sum of dextramer percentages in a single individual) (right graph). (C) Percentage of dextramer^+^CD8^+^ cells in 14 HDs, 9 Rs to etanercept, and 6 NRs (each symbol represents the percentage of a single dextramer^+^CD8^+^ cell population) (left graph); sum of the percentages of all dextramer^+^CD8^+^ T cells detected in the single HD, R, and NR (each symbol represents the sum of dextramer percentages in a single individual) (right graph). Analyses were performed at time 0 (before the start of therapy). Statistical analysis was performed with the Mann-Whitney test. **P < 0.001; ***P < 0.0001. (D) ROC curve analyses for R and NR patients. AUC = area under receiver operating characteristic curve. * = predictive value of positive test. # = predictive value of negative test.

To explore the possibility that the frequencies of CD8^+^ T cells specific to AE, as detected by ELISPOT assay, did not differ between Rs and NRs (Fig 1A) because our ELISPOT system identified only IFN-**γ** ^+^ cells, we conducted further testing. In particular, we used the class I molecule multimer technology, thereby allowing the entire AE-specific CD8^+^ T cell population to be counted with the same epitope specificity, irrespective of their differentiation phase, as well as T cells with an “exhaustion phenotype,” representing the reducing capacity of cells to perform effector functions [27]. We enumerated AE-specific CD8^+^ T cells in the peripheral blood of 15 HLA-A2^+^ RA patients submitted to etanercept (of whom 9 would be Rs and 6 would be NRs) by using dextramers of HLA-A*0201 molecules complexed to ACTB_266-274_, MYH_9478-486_, MYH_9741-749_, VIME_78-87_, or VIME_225-233_ peptides (Fig 2).

**Fig 3:**
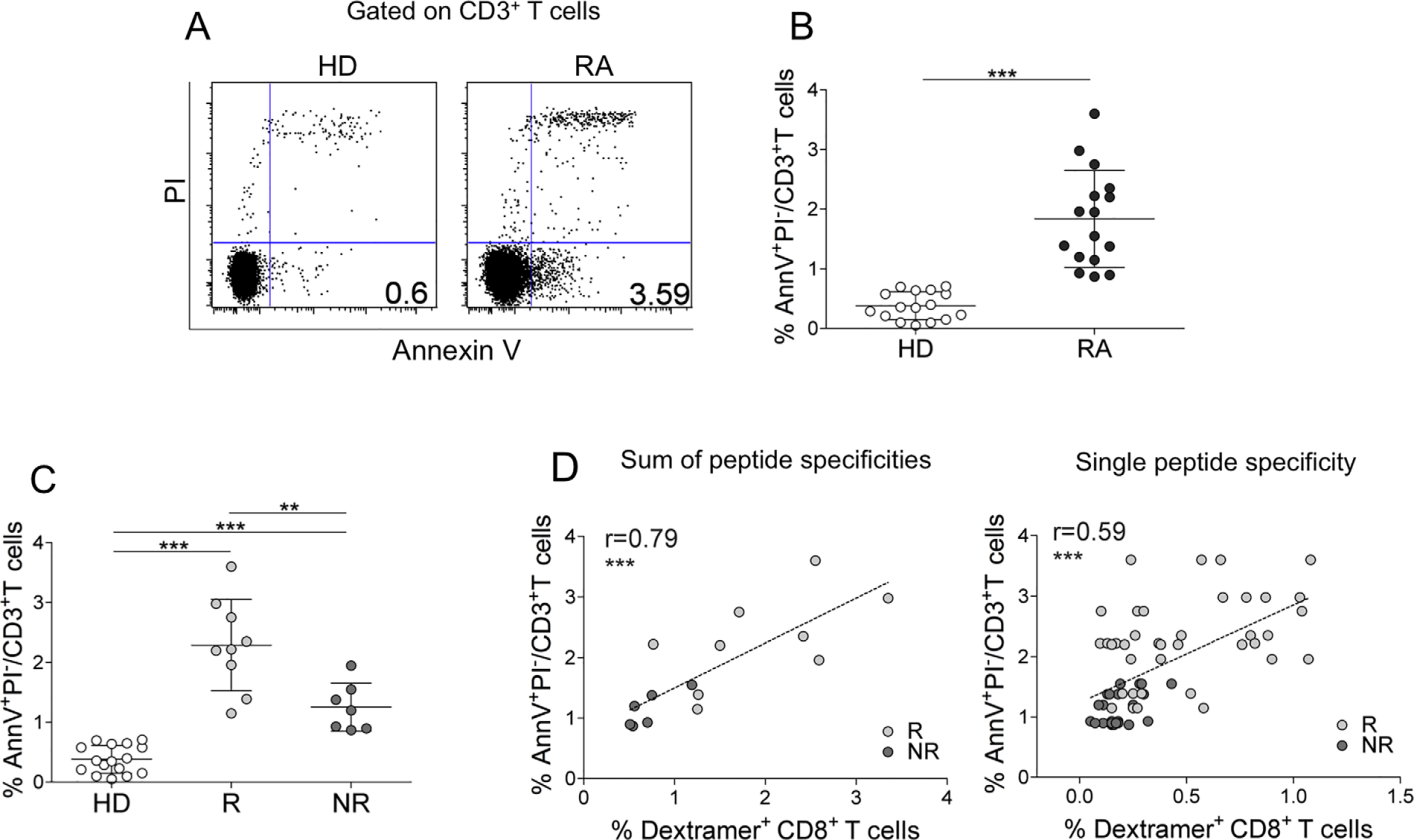
AE-specific CD8^+^ T cells correlate with circulating apoptotic T cells in RA patients (A-D). (A) Representative flow cytometry analysis of double-stained Annexin V/PI^+^ cells in CD3^+^ T cells from an HD or an RA patient. The percentage of Annexin V^+^/PI^-^cells is indicated in the appropriate quadrant. (B) Percentage of circulating early (Annexin V^+^/PI^-^) apoptotic T cells in 16 HDs and 16 RA patients studied. Statistical analysis was performed with the Mann-Whitney test. ***P < 0.0001. (C) Percentage of circulating early (Annexin V^+^/PI^-^) apoptotic T cells in all HDs and RA patients (9 Rs or 7 NRs to TNF-α inhibitor therapy) studied. Statistical analysis was performed with the Mann-Whitney test. **P < 0.001; ***P < 0.0001. (D) Correlation between circulating early apoptotic T cells and the sum of percentages of dextramer^+^CD8^+^ cells specific to a single epitope from the single R or NR (each symbol represents a single patient) (Spearman correlation analysis) (left graph); correlation between circulating early apoptotic T cells and percentage of all dextramer^+^CD8^+^ cells specific to a single epitope from R or NR (each symbol represents dextramer^+^CD8^+^ cells specific to a single AE) (right graph) (Spearman correlation analysis). Analyses were performed at time 0 (before the start of therapy).

Control dextramers complexed to a non-natural irrelevant peptide were unable to stain CD8^+^ T cells in all samples tested (data not shown). All patients presented frequencies of peripheral dextramer^+^CD8^+^ T cells significantly higher than HDs, in terms of both responsiveness and magnitude (Fig 2A and 2B). Amazingly, the total frequencies of AE-specific CD8^+^ T cells, as detected by dextramers, were significantly higher in Rs than in NRs to etanercept therapy at time 0 (Fig 2C). Similarly, the AE-specific CD8^+^ T cells frequency was significantly higher in Rs than in NRs to adalimumab therapy at time 0 (S2 Fig) To evaluate the discriminatory accuracy of AE-specific CD8^+^ T cells, ROC analysis was performed. When a comparison was made between Rs and NRs, the AUC was 0.82 (95% CI = 0.722–0.925, *P* < 0.0001) (Fig 2D). By contrast, no differences in DAS28-ESR, DAS28-CRP, age, disease duration, and presence of ACPAs between Rs and NRs were observed at time 0 (S1 Table and S3 Fig). The percentage of early apoptotic T cells (as detected by Annexin V staining) circulating in PBMCs was significantly more elevated in total patients than in HDs (Fig 3A and 3B), as well as (more important) significantly more elevated in Rs than in NRs (Fig 3C).

**Fig 4:**
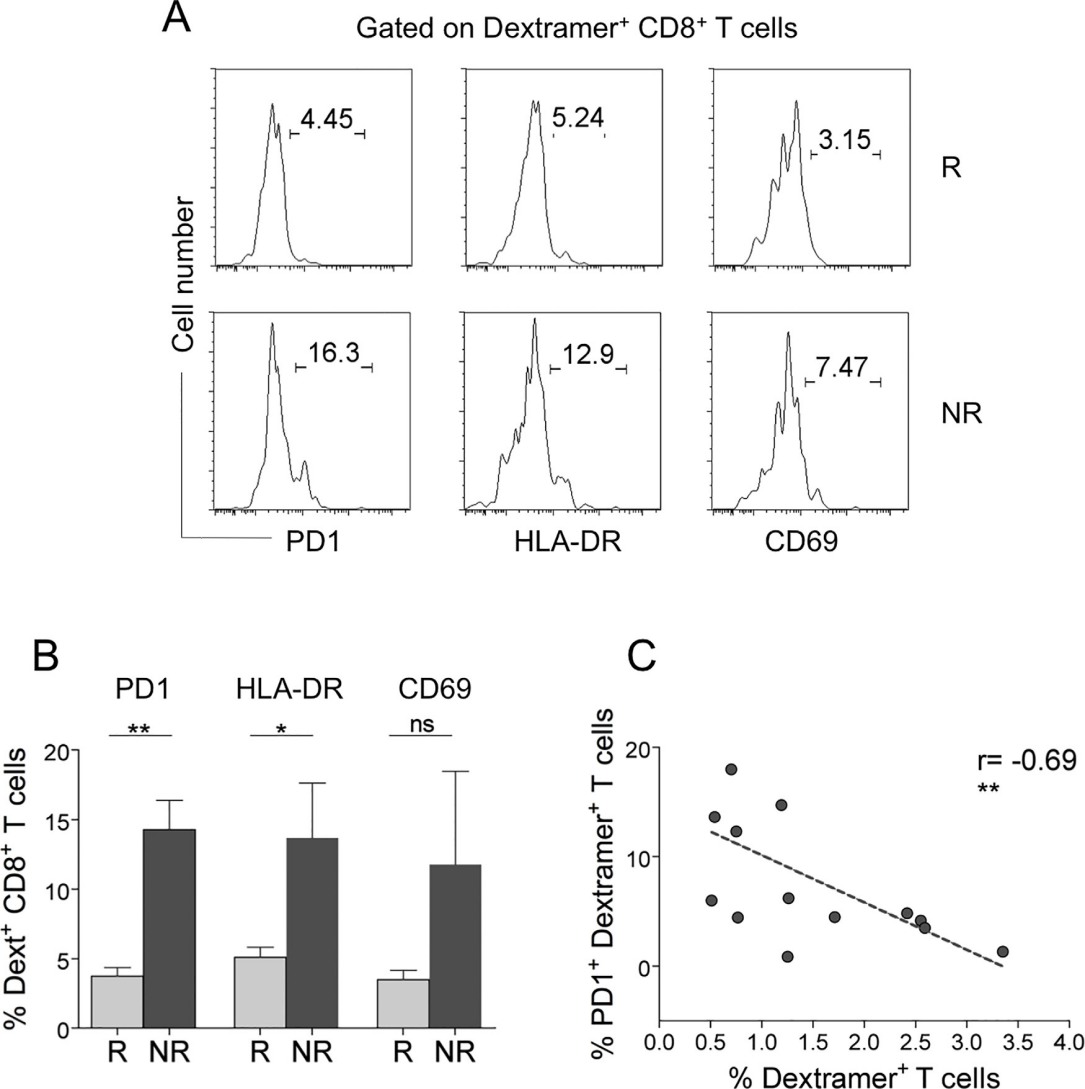
Expression of activation markers by AE-specific CD8^+^ T cells in Rs and NRs (A-C). (A) Representative flow cytometry analysis of PD-1, HLA-DR, and CD69 in AE-specific dextramer^+^CD8^+^ T cells from an R and an NR. (B) Percentage of AE-specific dextramer^+^CD8^+^ T cells expressing PD-1, HLA-DR, or CD69 in all Rs and NRs studied. Statistical analysis was performed with the Mann-Whitney test. *P < 0.01; **P < 0.001; ns = not significant. (C) Correlation between AE-specific dextramer^+^CD8^+^ T cells expressing PD-1 and total AE-specific dextramer^+^CD8^+^ T cells (Spearman correlation analysis). **P < 0.001. Analyses were performed at time 0 (before the start of therapy).

Notably, it directly correlated with the frequency of AE-specific CD8^+^ T cells (Fig 3D), suggesting a possible cause-and-effect relationship between the two events. A significant proportion of these AE-specific CD8^+^ T cells expressed late activation markers (e.g., HLA-DR and PD-1), indicating that they are experienced T cells (Fig 4A and 4B). However, in contrast to the total AE-specific CD8^+^ T cells that were significantly higher in Rs (see Fig 2), the experienced AE-specific CD8^+^ T cells (and, in particular, those expressing the PD-1 exhaustion marker) were represented more in NRs than in Rs (Fig 4A and 4B).

**Fig 5:**
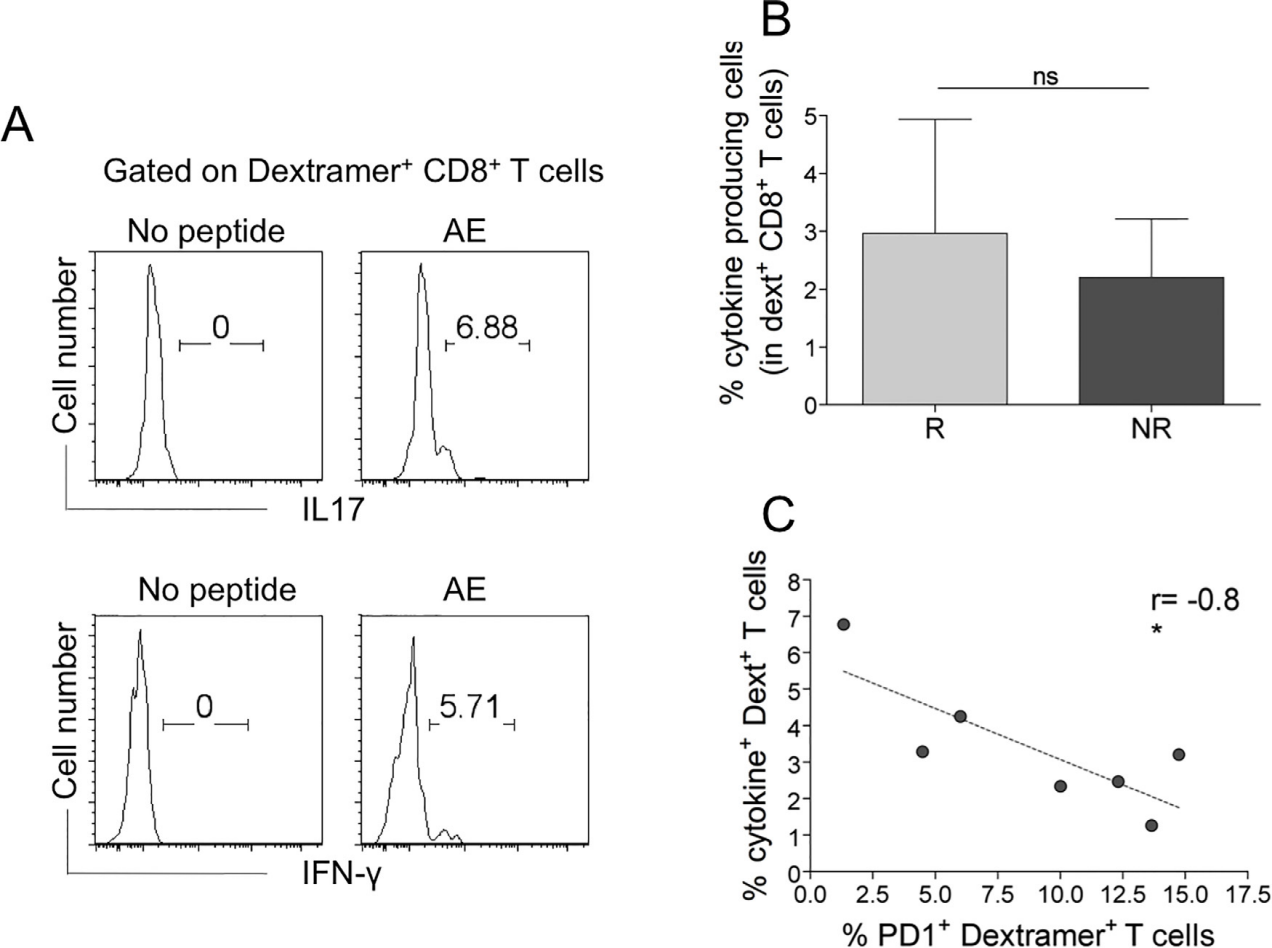
AE-specific dextramer^+^CD8^+^ T cells specifically producing inflammatory cytokines inversely correlate with PD-1^+^dextramer^+^CD8^+^ T cells (A-C). (A) Representative flow cytometry analyses of cells producing IL-17 or IFN-**γ** in dextramer^+^CD8^+^ T cells in response to a relevant AE pool (5 peptides). (B) Percentage of cytokine-producing cells in dextramer^+^CD8^+^ T cells from Rs and NRs. Statistical analysis was performed with the Mann-Whitney test. ns = not significant. (C) Correlation between cytokine-producing dextramer^+^CD8^+^ T cells and those expressing PD-1 (Spearman correlation analysis). Analyses were performed at time 0 (before the start of therapy).

Importantly, PD-1 expression inversely correlated with the frequencies of the total AE-specific CD8^+^ T cells (Fig 4C), a result suggesting that the inhibitory PD-1 molecule plays a role in tempering T cell survival/expansion, particularly in NRs showing the frequencies of these cells to be significantly lower than in Rs at time 0 (see Fig 2). To validate the antigen specificity of AE-specific (dextramer^+^) CD8^+^ T cells, we analyzed their capacity to produce inflammatory cytokines (IFN-**γ**, IL-17) within a few hours of contact with the relevant peptides and optimal concentrations of anti-CD28 mAb, which served as a surrogate costimulatory signal. Undetectable cytokine production was observed when AE-specific CD8^+^dextramer^+^ T cells of 20 HLA-A2^+^ HDs were stimulated with this procedure (data not shown). Notably, AE-specific CD8^+^dextramer^+^ T cells produced moderate amounts of IFN-**γ** or IL-17 in response to the relevant epitopes *ex vivo*, providing evidence that they were effectively antigen-specific TEM cells (Fig 5).

Although the level of these responses was not significantly different between Rs and NRs at time 0 (Fig 5A and 5B), they were inversely correlated with the percentage of AE-specific CD8^+^ T cells expressing PD-1 (Fig 5C), which were represented more in NRs than in Rs (see Fig 4B).

### Follow-Up of AE-Specific CD8^+^ T Cells in RA Patients Treated with TNF-α Inhibitors

**Fig 6:**
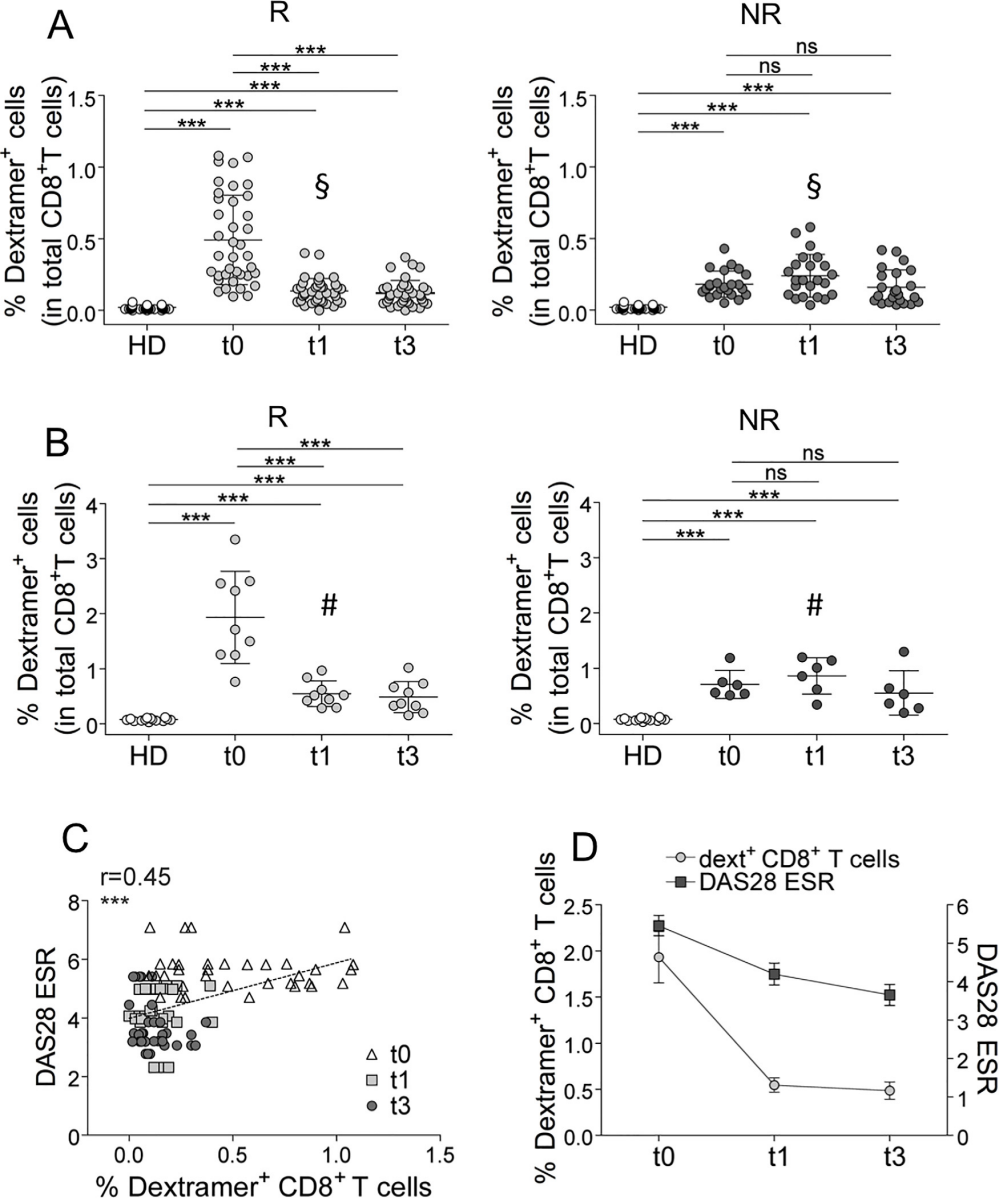
Time course analyses performed longitudinally throughout the follow-up in TNF-α inhibitor-treated patients (A-D). (A) Percentages of the single AE-specific dextramer^+^CD8^+^ T cells determined before (t0) the start of TNF-**α** inhibitor therapy and after 1 month (t1) and 3 months (t3) from the start of TNF-**α** inhibitor therapy in Rs and NRs (each symbol represents a single AE-specific dextramer^+^CD8^+^ T cell population). Statistical analysis was performed with the Wilcoxon matched pairs test. ***P < 0.0001; ns = not significant. The § symbol indicates the percentages of single AE-specific dextramer^+^CD8^+^ T cells that were compared between Rs and NRs at the t1 (**P < 0.001). (B) Sum of percentages of the single-specific dextramer^+^CD8^+^ T cells in single R and NR (each symbol represent a single patient). Statistical analysis was performed with the Wilcoxon matched pairs test. ***P < 0.0001; ns = not significant. The # symbol indicates the percentages of single AE-specific dextramer^+^CD8^+^ T cells that were compared between Rs and NRs at the t1 (**P < 0.001) (C) Correlation between DAS28-ESR and percentage of AE-specific dextramer^+^CD8^+^ T cells in Rs (Spearman correlation analysis). (D) Parallel follow-up of AE-specific dextramer^+^CD8^+^ T cell analyses and DAS28-ESR at t0, t1, and t3 from the start of TNF-**α** inhibitor therapy in Rs.

Time course analyses were then performed in patients treated with TNF-**α** inhibitors. They revealed that ELISPOT responses, which were equally represented in Rs and NRs at time 0 (see Fig 1), significantly declined only in Rs (S4 Fig); however, they did not correlate with the decline of disease activity (data not shown). This finding, together with the finding that the IFN-**γ** ^+^ ELISPOT assay did not display any predictive value (no difference was shown between Rs and NRs at time 0 [see Fig 1]), led us to monitor the frequencies of all the AE-specific CD8^+^ T cell populations with the same epitope specificity, irrespective of their differentiation phase (dextramer^+^ cells), so as to verify if they may provide more sensitive information during the time course analyses. The frequencies of AE-specific (dextramer^+^) CD8^+^ T cells (predicting the response to TNF-**α** inhibitor therapy [see Fig 2]) dropped in a significant fashion since the first month of therapy in Rs, but not in NRs (Fig 6A and 6B), and in a manner considerably more sensitive than that observed in the ELISPOT assay.

It is important to note that the AE-specific CD8^+^ T cell frequency in Rs after 1 month of therapy decreased at a level significantly lower than the corresponding frequencies in NRs (Fig 6A and 6B). Importantly, the decrease of the AE-specific CD8^+^ T cells in Rs, but not in NRs (not shown), was related to the reduction of clinical parameters (e.g., DAS28-ESR) (Fig 6C and 6D), strongly suggesting a relationship between these cells and the immunopathology and chronic evolution of RA. Notably, the difference between Rs and NRs was also confirmed at the level of the frequency of CD8^+^ T cells specific to a single peptide (S5 Fig).

## Discussion

Here we demonstrate for the first time that frequency, multispecificity, and magnitude of CD8^+^ T cells directed to AEs were significantly higher in RA patients compared with HDs, indicating that they may contribute to RA pathogenesis, as well as previously demonstrated in various immunopathology conditions [16,22]. Amazingly, the frequencies of AE-specific CD8^+^ T cells (as detected by dextramers) were significantly higher in Rs than in NRs at time 0 (before the start of therapy), as indicated by the significant ROC sensitivity and specificity, and correlated with the disease activity only in Rs. This, together with the finding that no clinical criterion (including DAS28-ESR, DAS28-CRP, and ACPA) was capable of discriminating Rs and NRs, suggests that the frequency of AE-specific CD8^+^ T cells represents a unique biomarker predicting the response to TNF-**α** inhibitor therapy in RA patients. However, these T cell responses to AEs are unlikely to represent the first event that initiates RA pathology. Indeed, our previous studies demonstrated that these responses require stimulation by apoptotic cells that derive from preexisting activated T cells, such as the virus-specific T cells that are generated during HIV or HCV infections [16,22]. We can envisage that in RA, the primary activated T cells may be specific for disease-specific self-antigens (e.g., citrullinated epitopes) or even some putative pathogen (possibly correlated with RA) that starts the immunological cascade leading to RA, and, consequently, may provide the first boost of apoptotic antigens to AE-specific T cells [15]. According to this scenario, the frequencies of AE-specific T cell responses were directly correlated with the percentage of circulating apoptotic T cells, suggesting that the emergence of AE-specific CD8^+^ T cells is selectively related to the numbers of apoptotic T cells in RA patients. AE-specific CD8^+^ T cells may strongly contribute to amplifying and sustaining RA damage through a vicious cycle providing continuous waves of apoptotic antigens upon performing their pro-inflammatory activity, as indicated by their correlation with the disease activity. The finding that the same responses are operative in various chronic infections (i.e., HIV or HCV [16,22]) or in autoimmune disorders, such as MS [23], suggests that they may support a general mechanism of CIA in several immune-mediated diseases. By contrast, evidence showing the inability of pancreatic **β**-cell apoptosis to provoke the development of dominant AE-specific CD8^+^ T cell responses in type 1 diabetes in mice [28] could be attributed to various non-mutually exclusive possibilities. One possibility is that apoptotic **β** cells are CD40L^-^ epithelial cells, which are generally unable to initiate efficient cross-priming of AE-specific T cells (as CD40L^+^ apoptotic T cells do [22,23]). Another possibility is that the generation and maintenance of functional AE-specific responses likely require robust or long-term inflammatory responses that provide sustained apoptosis of activated T cells, particularly occurring during human acute or chronic inflammatory diseases.

An important question that arises from our finding is why the frequencies of AE-specific CD8^+^ T cells are significantly more elevated in Rs than in NRs before the start of therapy. An attractive hypothesis is that AE-specific CD8^+^ T cells are more prone to PD-1-dependent exhaustion in NRs. This hypothesis is based on the following findings: (i) PD-1 expression in AE-specific CD8^+^dextramer^+^ cells is significantly higher in NRs than in Rs, and correlates with the decreased expansion of these cells; (ii) the percentage of AE-specific CD8^+^dextramer^+^ cells producing cytokines in response to the relevant AE inversely correlates with the PD-1 expression. This mechanism has been defined in various chronic viral and autoimmune diseases [16,22,23]. Recently, a strict relationship between sustained type I IFN (IFN-I) signaling and PD-1 overexpression on T cells, resulting in the establishment of a low level of CIA and decreased effector functions by virus-specific T cells, has been demonstrated in the mouse model of persistent lymphocytic choriomeningitis virus infection [29,30]. With the relative differences having been taken into account, the low level of CIA (likely attributed to upregulation of PD-1 expression) shown in NRs might be at a subthreshold level so as to be susceptible to TNF-**α** inhibitor therapy, which consequently might not restrain the chronic low-level disease progression. By contrast, Rs with a higher level of CIA would be more susceptible to TNF-**α** inhibitor therapy, ultimately leading to control of disease activity. It will be of interest to verify whether a genetic background predisposing to autoimmunity harbors variants that may foster this mechanism. Notably, we studied RA patients who were NRs to a previous therapy regime with methotrexate in combination (or not) with other anti-inflammatory/immunosuppressive drugs (see S1 Table). Further studies are required to determine if AE-specific CD8^+^ T cells distinguish different treatment-naïve patient populations (Rs or NRs) and/or if methotrexate or other immunosuppressive drugs play a role in the development of these responses in patients with a particular genetic signature that would contribute to establishing their profile.

Our previous data clearly demonstrated that caspase cleavage of apoptotic antigens is required to activate the related CD8^+^ T cells by cross-presentation, indicating that these autoreactive CD8^+^ T cells may contribute to immunopathology through the production of pro-inflammatory cytokines upon cross-presentation of a huge number of apoptotic cells that infiltrate inflamed tissues [31] rather than by the direct killing of target cells [15,16]. In addition, other mechanisms may contribute to establishing chronic immunopathological processes. Recently, active Epstein-Barr virus (EBV) infection has been demonstrated within ectopic lymphoid structures in the RA synovium in association with local differentiation of ACPA-reactive B cells [32]. Moreover, several independent memory T cells (e.g., those that are specific to a multitude of pathogens normally circulating in the lymphoid tissues), which are stimulated in a bystander fashion, can be recruited in an inflammatory site where they can perform effector functions [2,8,33,34].

However, irrespective of their possible pathogenic role, the finding that the total AE-specific CD8^+^ T cells are significantly more elevated in Rs than in NRs to etanercept since time 0 strongly suggests that these cells may have the potentiality for predicting patients who will (or will not) benefit from TNF-**α** inhibitor therapy. This possibility appears to be clinically valuable and appropriate for budgeting, thus allowing a preemptive submission of NRs to different boDMARDs. In this context, ongoing experiments validate the usage of AE-specific CD8^+^ T cells as biomarkers predicting the response to a different TNF-**α** inhibitor, such as adalimumab. The finding that the detection of CD8^+^ T cells specific to a single AE was sufficient to discriminate Rs and NRs suggests that only one of them can be exploited as a potential predictive biomarker of response to TNF-**α** inhibitor therapy. Further studies involving a larger study population are needed to ascertain whether AE-specific CD8^+^ T cells could be exploited as a biomarker predicting response to alternative boDMARDs.

## Supporting Information

S1 Table Study population. (DOCX)

S2 Table HLA-A2 binding peptides derived from apoptotic cell-associated proteins (Pools 1–4). (DOCX)

S3 Table HLA-A2 binding peptides derived from apoptotic cell-associated proteins (Pools 5–8). (DOCX)

S4 Table HLA-A2 binding peptides derived from apoptotic cell-associated proteins (Pools 9–12). (DOCX)

S1 Fig DAS28 ESR score in R and NR. Patients were assessed for overall disease activity using the DAS28, and categorized in Rs or NRs according to the EULAR criteria 6 months after the start of treatment. An improvement of the DAS28 >0.6 was considered a response to therapy. Statistical was performed with Paired t test analysis. ***P<0.0001. ns = not significant.
(TIF)

S2 Fig AE-specific CD8^+^ T cells predict RA patients who will (or will not) benefit from adalimumab therapy. Percentage of dextramer^+^CD8^+^ cells in 14 HDs, 2 Rs to adalimumab, and 4 NRs (each symbol represents the percentage of a single dextramer^+^CD8^+^ cell population). Statistical analysis was performed with the Mann-Whitney test. **P < 0.001; ***P < 0.0001.
(TIF)

S3 Fig Clinical criteria do not discriminate Rs and NRs. DAS28 ESR values (A), DAS28 CRP values (B), and RA duration (months) (C), before the start of TNF-inhibitor therapy, in R and NR. Statistical analysis was performed with the Mann-Whitney test. ns = not significant.
(TIF)

S4 Fig Follow-up of IFN-γ^+^ CD8^+^ T cells specific for AEs as detected by ELISPOT. Sum of IFN-**γ** ^+^ cell spots formed in response to AE pools, analyzed in R and NR at t0, t1, and t3 from the start of anti-TNF-α therapy. Statistical analysis performed with Wilcoxon matched pairs test *P<0.01; **P<0.001; ***P<0.0001. ns = not significant.
(TIF)

S5 Fig Time course analyses of the dextramer^+^CD8^+^ T cells specific for the single AE. Percentage of dextramer^+^CD8^+^ T cells specific for the single AE indicated, in R and NR. Analyses were performed at t0, t1, and t3 from the start of TNF-**α** inhibitor therapy. Statistical analysis was performed with Wilcoxon matched pairs test *P <0.01; **P<0.001; ***P <0.0001. ns = not significant.
(TIF)
