# Supplementary material for: CD8+ T Cells Specific to Apoptosis-Associated Antigens Predict the Response to Tumor Necrosis Factor Inhibitor Therapy in Rheumatoid Arthritis
Source: PLoS One. 2015 Jun 10;10(6):e0128607. doi: 10.1371/journal.pone.0128607 (PMC4465029; doi:10.1371/journal.pone.0128607)
Supplement: S1 Table — (DOCX) [file pone.0128607.s001.docx]

|  | **Gender** | **Age** | **Disease Duration**  **(months)** | **DMARDS*** | **ACPA**** | **DAS28 ESR** | **Clinical**  **outcome** |
| --- | --- | --- | --- | --- | --- | --- | --- |
|  |  |  |  |  |  |  |  |
| 1 | F | 59 | 18 | MTX, HCQ | neg | 3.85 | Non Responder |
| 2 | F | 59 | 84 | MTX | pos | 6.01 | Non Responder |
| 3 | F | 54 | 72 | MTX, HCQ, SSZ | pos | 5.82 | Responder |
| 4 | F | 36 | 48 | MTX, HCQ | neg | 1.26 | Non Responder |
| 5 | F | 38 | 16 | MTX, HCQ | neg | 4.7 | Responder |
| 6 | F | 68 | 120 | MTX, HCQ | neg | 5.44 | Non Responder |
| 7 | M | 51 | 18 | MTX, HCQ, SSZ | pos | 5.08 | Responder |
| 8 | F | 43 | 60 | MTX | pos | 5.02 | Non Responder |
| 9 | F | 62 | 84 | MTX, LEF | neg | 4.91 | Non Responder |
| 10 | F | 69 | 84 | MTX | pos | 5.65 | Responder |
| 11 | F | 48 | 240 | MTX, HCQ | neg | 5.18 | Responder |
| 12 | F | 60 | 6 | MTX | neg | 7.08 | Responder |
| 13 | F | 53 | 48 | MTX | neg | 5.44 | Responder |
| 14 | F | 56 | 156 | MTX | pos | 5.84 | Responder |
| 15 | F | 66 | 216 | MTX, HCQ | neg | 4.74 | Non Responder |
| 16 | F | 44 | 216 | SO, CsA, MTX, HCQ | pos | 4.32 | Responder |

**S1 Table. Study population**

* Disease Modifying Anti-Rheumatic Drugs

**Anti-citrullinated protein autoantibodies
