# Supplementary material for: CD8+ T Cells Specific to Apoptosis-Associated Antigens Predict the Response to Tumor Necrosis Factor Inhibitor Therapy in Rheumatoid Arthritis
Source: PLoS One. 2015 Jun 10;10(6):e0128607. doi: 10.1371/journal.pone.0128607 (PMC4465029; doi:10.1371/journal.pone.0128607)
Supplement: S2 Table — (DOCX) [file pone.0128607.s002.docx]

| **S2 Table: HLA-A2 binding peptides derived from**  **apoptotic cell-associated proteins (Pools 1-4)** | | | | | |
| --- | --- | --- | --- | --- | --- |
| **Pool** | **Organism** | **Protein** | **1^st^ pos°** | **Sequence** | **Length** |
| **1** | Human | ACT | 131 | AMYVAIQAV | 9 |
|  | Human | ACT | 319 | ALAPSTMKI | 9 |
|  | Human | ACT | 266 | FLGMESCGI | 9 |
|  | Human | ACT | 312 | RMQKEITAL | 9 |
|  | Human | ACT | 348 | SLSTFQQMWI | 10 |
|  | Human | ACT | 46 | GMGQKDSYV | 9 |
| **2** | Human | ROK | 154 | SLAGGIIGV | 9 |
|  | Human | ROK | 67 | ALRTDYNASV | 10 |
|  | Human | ROK | 193 | VLIGGKPDRV | 10 |
|  | Human | ROK | 209 | ILDLISESPI | 10 |
|  | Human | ROK | 122 | QLPLESDAV | 9 |
| **3** | Human | LAM 1 | 496 | TIWAANAGV | 9 |
|  | Human | LAM 1 | 41 | RLAVYIDKV | 9 |
|  | Human | LAM 1 | 301 | SLSSQLSNL | 9 |
|  | Human | LAM 1 | 361 | QLLDVKLAL | 9 |
|  | Human | LAM 1 | 291 | ELMESRMRI | 9 |
|  | Human | LAM 1 | 355 | QLNDYEQLL | 9 |
|  | Human | LAM 1 | 388 | KLSPSPSSRV | 10 |
|  | Human | LAM 1 | 488 | VLKAGQTVTI | 10 |
|  | Human | LAM 1 | 378 | KLLEGEEERL | 10 |
| **4** | Human | MYH9 | 9 | YLYVDKNFI | 9 |
|  | Human | MYH9 | 108 | GLIYTYSGL | 9 |
|  | Human | MYH9 | 111 | YTYSGLFCV | 9 |
|  | Human | MYH9 | 145 | EMPPHIYAI | 9 |
|  | Human | MYH9 | 186 | KVIQYLAYV | 9 |
|  | Human | MYH9 | 478 | QLFNKHTMFI | 9 |
|  | Human | MYH9 | 584 | WLMKNMDPL | 9 |
|  | Human | MYH9 | 653 | QLAKLMATL | 9 |
|  | Human | MYH9 | 111 | YTYSGLFCVV | 10 |

°= 1st amino acid position; ACT= actin cytoplasmatic 1; ROK= eterogeneous nuclear ribonucleoprotein K; LAM 1= Iamin B1; MYH9 = non muscle myosin.
