# Supplementary material for: CD8+ T Cells Specific to Apoptosis-Associated Antigens Predict the Response to Tumor Necrosis Factor Inhibitor Therapy in Rheumatoid Arthritis
Source: PLoS One. 2015 Jun 10;10(6):e0128607. doi: 10.1371/journal.pone.0128607 (PMC4465029; doi:10.1371/journal.pone.0128607)
Supplement: S3 Table — (DOCX) [file pone.0128607.s003.docx]

| **S3 Table: HLA-A2 binding peptides derived from**  **apoptotic cell-associated proteins (Pools 5-8)** | | | | | |
| --- | --- | --- | --- | --- | --- |
| **Pool** | **Organism** | **Protein** | **1^st^ pos** | **Sequence** | **Length** |
| **5** | Human | MYH9 | 424 | RMFRWLVLRI | 10 |
|  | Human | MYH9 | 478 | QLFMHTMFIL | 10 |
|  | Human | MYH9 | 302 | FLSNGHVTI | 9 |
|  | Human | MYH9 | 338 | GLLRVISGV | 9 |
|  | Human | MYH9 | 412 | FAIEALAKA | 9 |
|  | Human | MYH9 | 450 | ILDIAGFEI | 9 |
|  | Human | MYH9 | 733 | FMDGKQACV | 9 |
|  | Human | MYH9 | 741 | VLMIKALEL | 9 |
|  | Human | MYH9 | 1277 | KLQVELDNV | 9 |
| **6** | Human | MYH9 | 1843 | KLKDVLLQV | 9 |
|  | Human | MYH9 | 279 | YLLSGAGEHL | 10 |
|  | Human | MYH9 | 733 | FMDGKQACVL | 10 |
|  | Human | MYH9 | 1920 | KLRRGDLPFV | 10 |
|  | Human | MYH9 | 210 | QLLQANPIL | 9 |
|  | Human | MYH9 | 847 | MMAKEEELV | 9 |
|  | Human | MYH9 | 877 | QLMAEKLQL | 9 |
|  | Human | MYH9 | 1726 | RLEARIAQL | 9 |
|  | Human | MYH9 | 1793 | KLQEMEGTV | 9 |
| **7** | Human | MYH9 | 660 | TLRNTNPNFV | 10 |
|  | Human | MYH9 | 688 | VLDQLRCNGV | 10 |
|  | Human | MYH9 | 752 | NLYRIGQSKV | 10 |
|  | Human | MYH9 | 248 | YIVGANIET | 9 |
|  | Human | MYH9 | 1540 | QLEELEDEL | 9 |
|  | Human | MYH9 | 161 | MMQDREDQSI | 10 |
|  | Human | MYH9 | 821 | KLRNWQWWRL | 10 |
|  | Human | MYH9 | 846 | EMMAKEEELV | 10 |
| **8** | Human | GDIS | 100 | VLKEGSEYRV | 10 |
|  | Human | GDIS | 186 | HLSWEWNLSI | 10 |
|  | Human | GDIS | 37 | EMDKDDESL | 9 |
|  | Human | GDIS | 51 | TLLGDGIVV | 9 |

°= 1st amino acid position; MYH9 = non muscle myosin; GDIS= rho GDP dissociation inhibitor 2.
