# Supplementary material for: CD8+ T Cells Specific to Apoptosis-Associated Antigens Predict the Response to Tumor Necrosis Factor Inhibitor Therapy in Rheumatoid Arthritis
Source: PLoS One. 2015 Jun 10;10(6):e0128607. doi: 10.1371/journal.pone.0128607 (PMC4465029; doi:10.1371/journal.pone.0128607)
Supplement: S4 Table — (DOCX) [file pone.0128607.s004.docx]

| **S4 Table: HLA-A2 binding peptides derived from**  **apoptotic cell-associated proteins (Pools 9-12)** | | | | | |
| --- | --- | --- | --- | --- | --- |
| **Pool** | **Organism** | **Protein** | **1^st^ pos** | **Sequence** | **Length** |
| **9** | Human | VIME | 176 | NLAEDIMRL | 9 |
|  | Human | VIME | 50 | SLIASSPGGD | 10 |
|  | Human | VIME | 68 | RLRSSVPGV | 10 |
|  | Human | VIME | 129 | ILLAELEQL | 9 |
|  | Human | VIME | 225 | SLQEEIAFL | 9 |
|  | Human | VIME | 78 | LLQDSVDFSL | 10 |
|  | Human | VIME | 79 | LQDSVDFSL | 9 |
|  | Human | VIME | 419 | SLNLRETNL | 9 |
|  | Human | VIME | 122 | FLEQQNKILL | 10 |
|  | Human | VIME | 370 | NMKEEMARHL | 10 |
| **10** | Human | PSA1 | 179 | FMECNLNEL | 9 |
|  | Human | PSA1 | 183 | NLNELVKHGL | 10 |
|  | Human | PSA1 | 175 | HMSEFMECNL | 10 |
|  | Human | PSA1 | 63 | ILHVDNHIGI | 10 |
|  | Human | PSA1 | 37 | GLKSKTHAV | 9 |
|  | Human | PSA1 | 110 | SLIGSKTQI | 9 |
|  | Human | PSA1 | 179 | FMECNLNELV | 10 |
|  | Human | PSA1 | 48 | ALKRAQSEL | 9 |
|  | Human | PSA1 | 76 | GLTADARLL | 9 |
| **11** | Human | PSA1 | 102 | PLPVSRLVSL | 10 |
|  | Human | PSA1 | 204 | DLTTKNVSI | 9 |
|  | Human | PSA1 | 45 | ELNGKNIEDV | 10 |
|  | Human | PSA1 | 55 | ELAAHQKKI | 9 |
|  | Human | PSA1 | 186 | ELVKHGLRAL | 10 |
|  | Human | PSA1 | 37 | GLKSKTHAVL | 10 |
|  | Human | PSA1 | 191 | GLRALRETL | 9 |
|  | Human | PSA1 | 55 | ELAAHQKKIL | 10 |
|  | Human | PSA1 | 97 | FVFDRPLPV | 9 |
| **12** | Human | RLA | 3 | YVASYLLAA | 9 |
|  | Human | RLA | 26 | ILDSVGIEA | 9 |
|  | Human | RLA | 3 | YVASYLLAAL | 10 |

°= 1st amino acid position; VIME= vimentin; PSA1= Proteasome component C2; RLA2= 60S acidic ribosomal protein P2.
