# Supplementary figures and images for: CD8+ T Cells Specific to Apoptosis-Associated Antigens Predict the Response to Tumor Necrosis Factor Inhibitor Therapy in Rheumatoid Arthritis
Source: PLoS One. 2015 Jun 10;10(6):e0128607. doi: 10.1371/journal.pone.0128607 (PMC4465029; doi:10.1371/journal.pone.0128607)

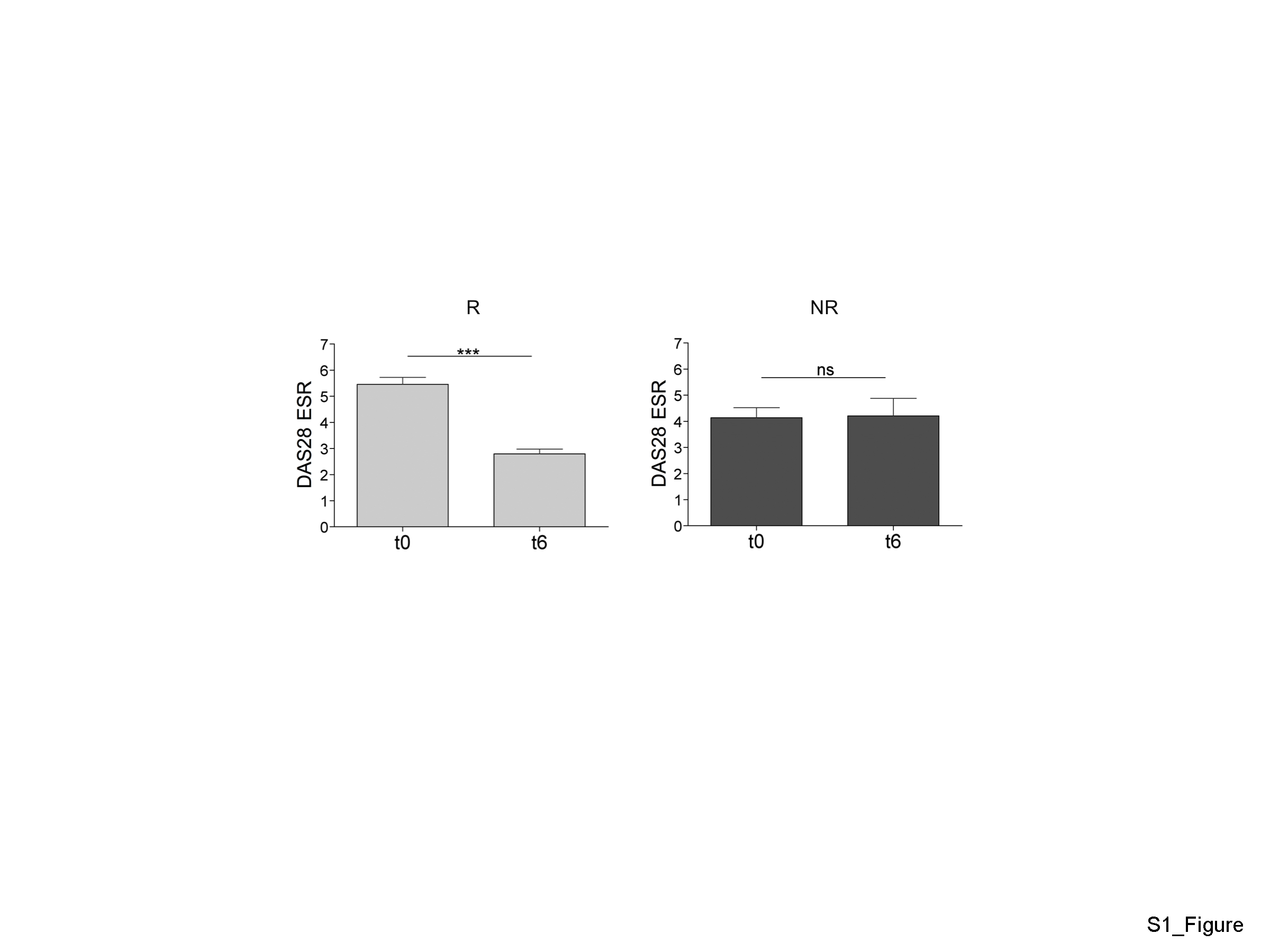

Supplement: S1 Fig — Patients were assessed for overall disease activity using the DAS28, and categorized in Rs or NRs according to the EULAR criteria 6 months after the start of treatment. An improvement of the DAS28 >0.6 was considered a response to therapy. Statistical was performed with Paired t test analysis. ***P<0.0001. ns = not significant. (TIF) [file pone.0128607.s005.tif]

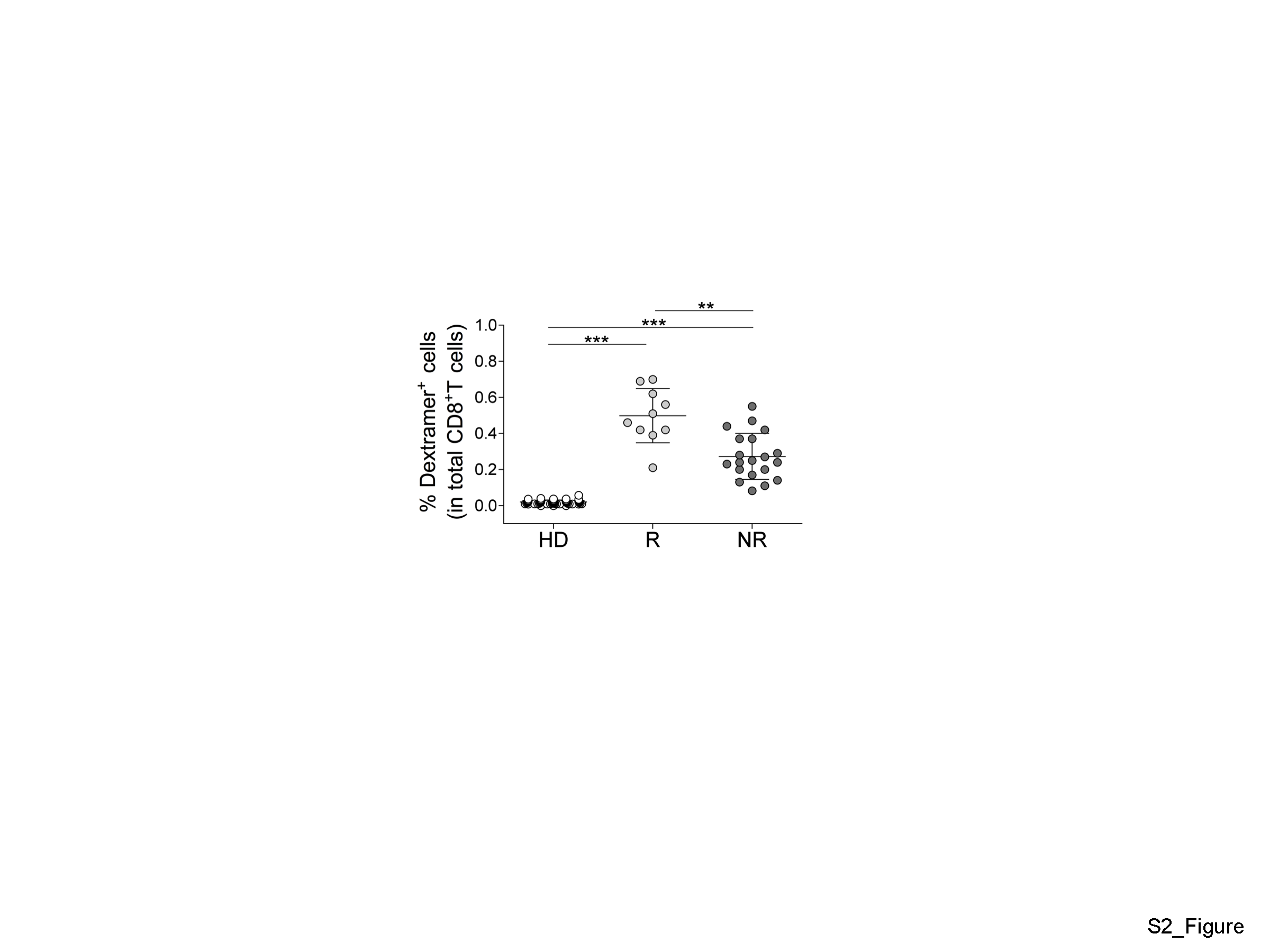

Supplement: S2 Fig — Percentage of dextramer+CD8+ cells in 14 HDs, 2 Rs to adalimumab, and 4 NRs (each symbol represents the percentage of a single dextramer+CD8+ cell population). Statistical analysis was performed with the Mann-Whitney test. **P < 0.001; ***P < 0.0001. (TIF) [file pone.0128607.s006.tif]

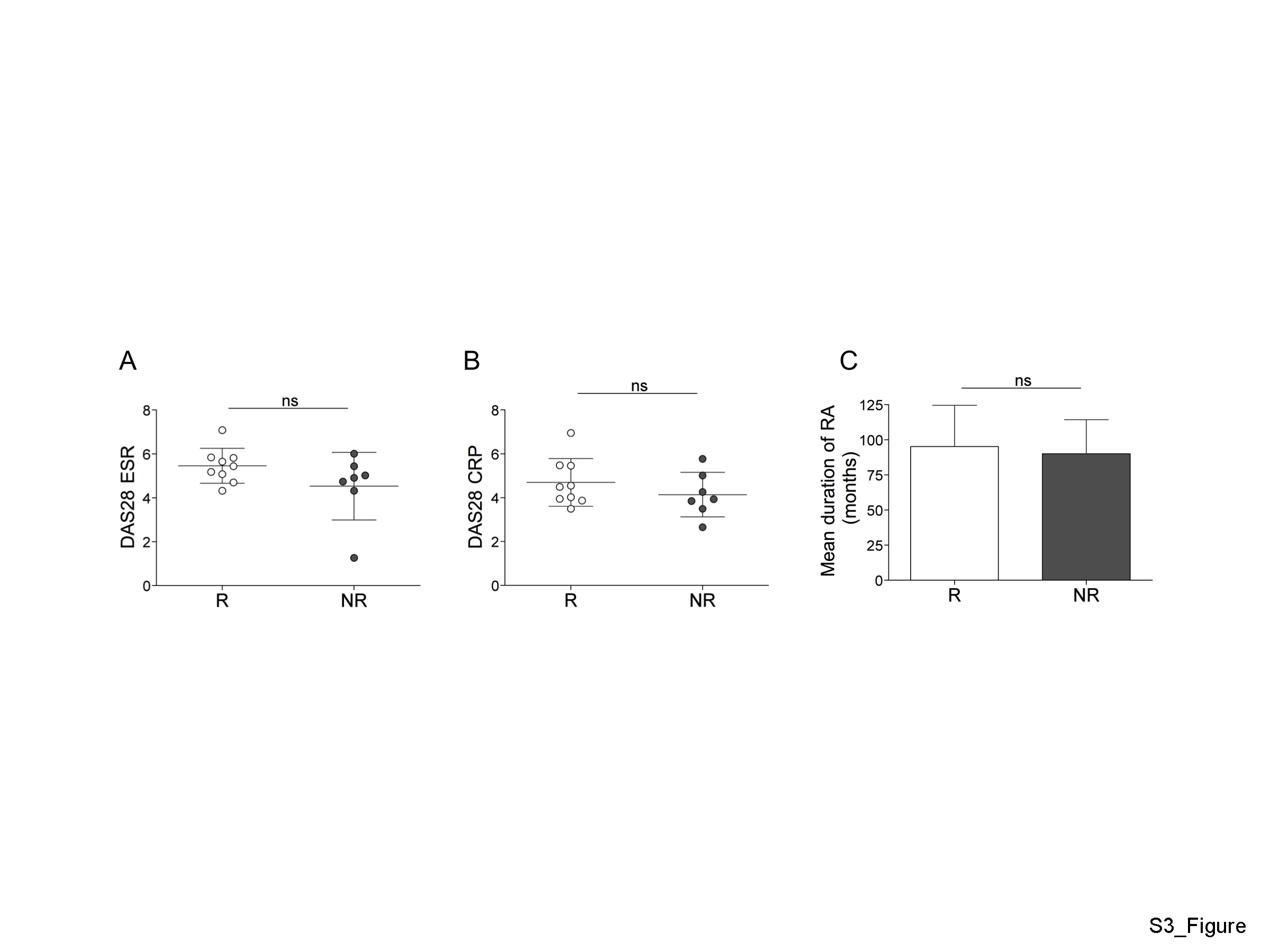

Supplement: S3 Fig — DAS28 ESR values (A), DAS28 CRP values (B), and RA duration (months) (C), before the start of TNF-inhibitor therapy, in R and NR. Statistical analysis was performed with the Mann-Whitney test. ns = not significant. (TIF) [file pone.0128607.s007.tif]

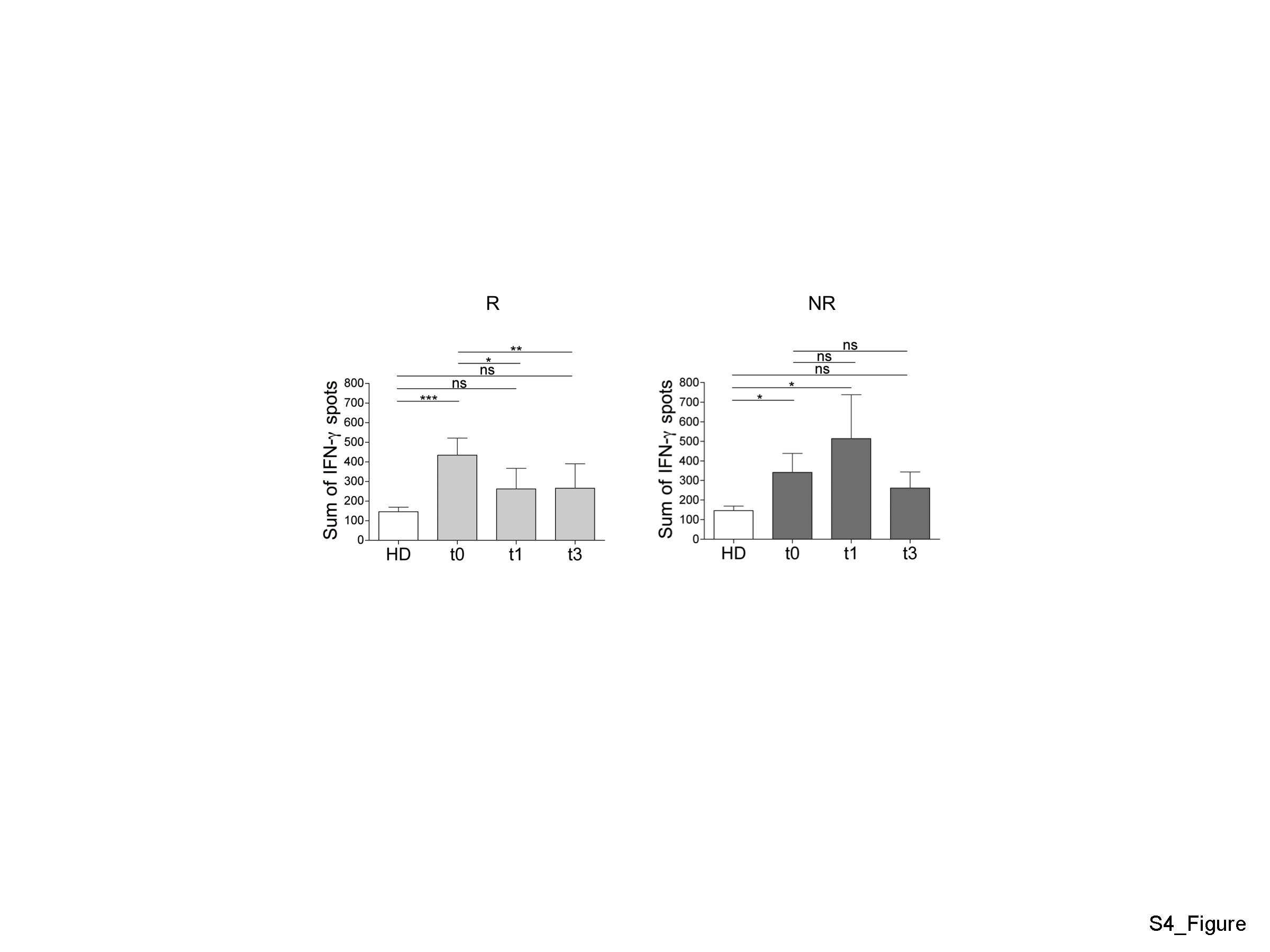

Supplement: S4 Fig — Sum of IFN-γ + cell spots formed in response to AE pools, analyzed in R and NR at t0, t1, and t3 from the start of anti-TNF-α therapy. Statistical analysis performed with Wilcoxon matched pairs test *P<0.01; **P<0.001; ***P<0.0001. ns = not significant. (TIF) [file pone.0128607.s008.tif]

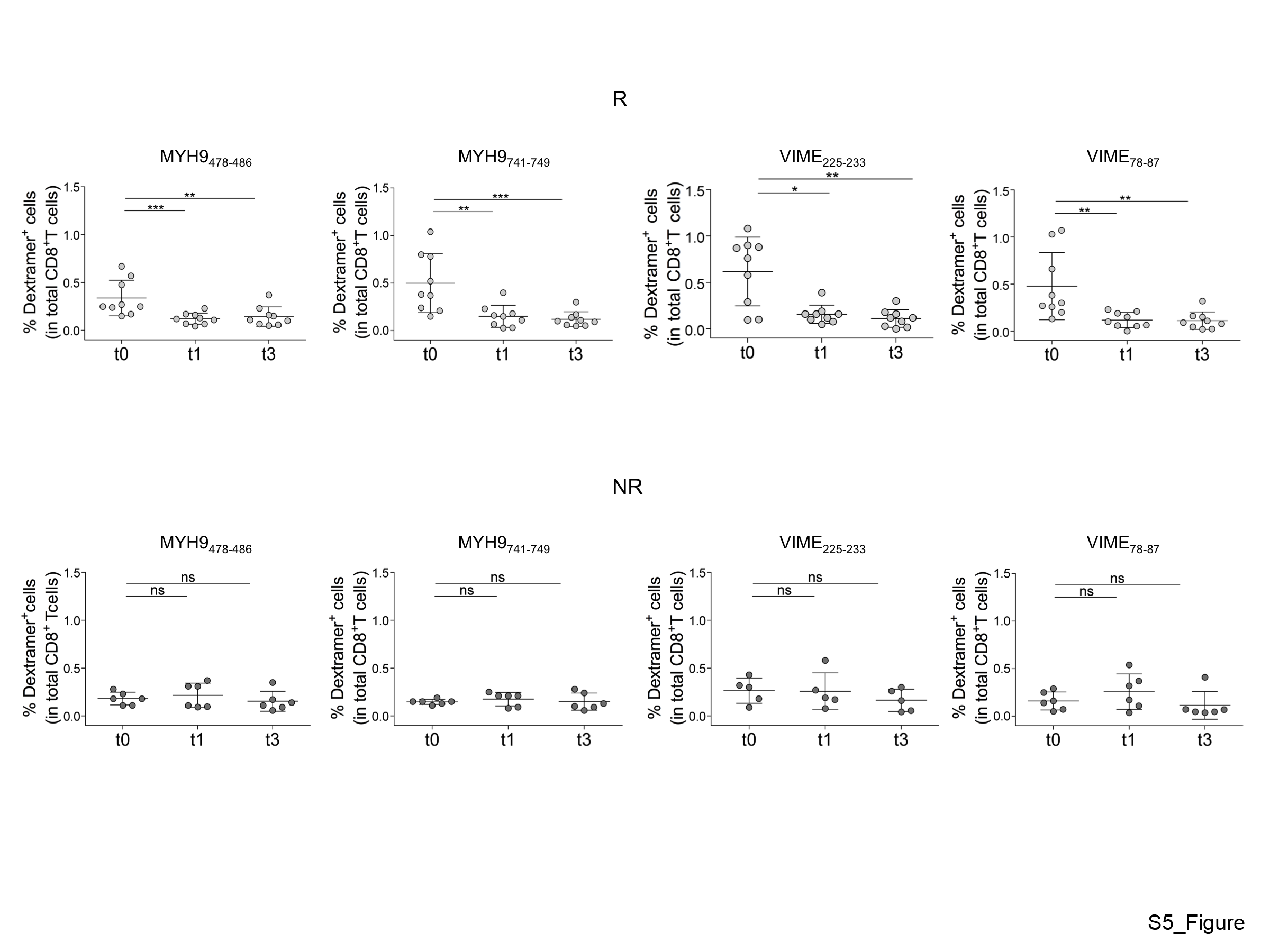

Supplement: S5 Fig — Percentage of dextramer+CD8+ T cells specific for the single AE indicated, in R and NR. Analyses were performed at t0, t1, and t3 from the start of TNF-α inhibitor therapy. Statistical analysis was performed with Wilcoxon matched pairs test *P <0.01; **P<0.001; ***P <0.0001. ns = not significant. (TIF) [file pone.0128607.s009.tif]
